# Research on *Trypanosoma cruzi* and Analysis of Inflammatory Infiltrate in Esophagus and Colon from Chronic Chagasic Patients with and without Mega

**DOI:** 10.1155/2012/232646

**Published:** 2011-10-30

**Authors:** Eliângela de Castro Côbo, Thales Parenti Silveira, Adilha Misson Micheletti, Eduardo Crema, Sheila Jorge Adad

**Affiliations:** ^1^Disciplina de Patologia Especial, Universidade Federal do Triângulo Mineiro (UFTM), Avenida Getúlio Guaritá 130, Abadia 38045-440 Uberaba, MG, Brazil; ^2^Disciplina de Cirurgia Digestiva, Universidade Federal do Triângulo Mineiro (UFTM), Avenida Getúlio Guaritá 130, Abadia 38045-440 Uberaba, MG, Brazil

## Abstract

To compare parasitism and inflammatory process in esophagus and colon from chronic chagasic patients, immunohistochemistry was carried out to research for *T. cruzi* and to evaluate the inflammatory infiltrate in the muscular and myenteric plexus in 39 esophagi (20 with and 19 without megaesophagus) and 50 colons (25 with and 25 without megacolon). The frequency of *T. cruzi* in megaesophagus was 20%, and in megacolon it was 4%. No amastigotes were found in organs without mega; considering the total of esophagi (with and without mega), the frequency of *T. cruzi* would be 10% and 2% in the colon. Myositis and ganglionitis were more frequent and intense in organs with mega compared to those without mega, and in esophagus compared to colon. Qualitatively, inflammatory infiltration in esophagus and colon, with or without mega, was similar, consisting predominantly of T lymphocytes (CD3+), scarce macrophages (CD68+), and rare B lymphocytes (CD20+).

## 1. Introduction

 The most frequent expression of the digestive form of Chagas disease, endemic in Central Brazil, is megaesophagus, followed by megacolon [1–3]. The organs with mega display a striking luminal enlargement and hypertrophy of the muscular layer. Inflammation is the basic pathological process of Chagas disease. The inflammatory response that follows the infection by *T. cruzi* is essential to the host resistance, but it is also responsible for the damage observed in Chagas disease [4]. The focal inflammation can be destructive not only for the infected cells, but also for the nonparasitized cells. Among the etiological components of megacolon, one of them is the immunologic nature [5]. Inflammatory infiltrate shows an important role in the pathogenesis of chronic chagasic myocarditis [6] and inflammatory lesions in the enteric nervous system, associated with a drastic reduction in the number of neurons, reinforcing clinical findings in chagasic megacolon/megaesophagus [7–10]. Reviewing the literature, we came across few studies of direct microscopic examination of *Trypanosoma cruzi* in chronically infected human tissues and we did not find studies evaluating both research on *T. cruzi* and inflammatory infiltration simultaneously in histological sections of esophagus and colon from chagasic patients with and without mega. The aim of this study was to evaluate comparatively the inflammatory process in muscular and myenteric plexus of esophagus and colon from chronic chagasic patients with and without mega, with the purpose of ascertaining whether there are similarities between the process in esophagus and colon and between cases with and without evident parasitism in the organ.

## 2. Material and Methods

We studied 50 segments of large intestine and 39 segments of esophagus from chronic chagasic patients obtained by surgery (16 colon and 4 esophagus) or autopsies performed at the Department of Surgical Pathology/Special Pathology from the Hospital of Universidade Federal do Triângulo Mineiro (UFTM), Uberaba, MG, Brazil. On 30 cases from autopsies, it was possible to use the esophagus and colon from the same individual. Of the 39 esophagus studied, 20 displayed mega and 19 had a normal diameter. From the 50 intestines assessed, 25 were carriers of megacolon and the other 25 had a normal caliber.

The diagnosis of Chagas infection was based on positivity of at least two of the three reactions in the blood and/or pericardial fluid: ELISA, passive hemagglutination and indirect immunofluorescence for *T. cruzi*. The age of the patients who formed the group of esophageal samples ranged from 34 to 84 years (55 ± 12) and colon from 22 to 84 years (55 ± 14). Regarding gender, 26 (67%) of the samples of esophagus and 30 (60%) of the samples of colons were males. In each case, an intestinal ring about 0.5cm “high” in the transition sigmoid-rectum and/or an esophageal ring 5cm distant from the cardia was collected. The rings were fixed in 4% formaldehyde and embedded in paraffin. Histological sections were stained according to the techniques of hematoxylin-eosin (HE) and Giemsa for global histological evaluation, assessing the occurrence, and classifying the intensity of myositis in the muscularis propria and ganglionitis in the myenteric plexus of the esophagus and colon. Immunohistochemistry (polymers technique) was performed for anti-CD20 (DAKO, dilution 1 : 600, clone: L26, Carpinteria, CA), anti-CD68 (DAKO, dilution 1 : 1000, clone: KP1, Carpinteria, CA), and anti-CD3 antibody (DAKO, Carpinteria, CA, dilution 1 : 400), in order to estimate the proportion of T and B lymphocytes and macrophages in inflammatory foci. The detection of *T. cruzi* was performed in 5 histological sections from each case, by immunohistochemistry, using anti-*T. cruzi* (rabbit serum obtained from the Faculty of Medicine of Triângulo Mineiro, dilution 1 : 600).

For statistical analysis the variables were evaluated using the GRAPHPAD INSTAT version 3.0. We used the Fisher's exact test to compare presence or absence of myositis and ganglionitis. For all tests the level of significance was 5% (*P* < 0.05).

Ethical approval for the study protocols was obtained from the research ethics committees of the Universidade Federal do Triângulo Mineiro (UFTM), Uberaba, MG, Brazil.

## 3. Results

### 3.1. *T. Cruzi*

**Figure 1:**
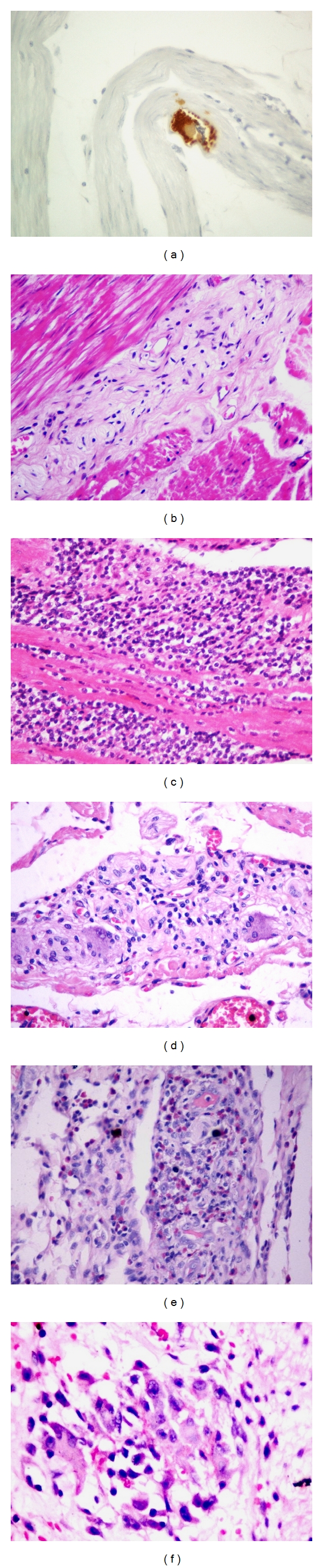
(a) Amastigotes forms of *T. cruzi* in muscular in case of megaesophagus (immunohistochemistry for *T. cruzi*—400x). (b) Aganglionosis and fibrosis in myenteric plexus in case of megaesophagus (HE—200x). (c) Severe chronic myositis in a case of megaesophagus (HE—400x). (d) Mild chronic ganglionitis in esophagus without mega (HE—400x). (e) Severe chronic ganglionitis surrounded by eosinophils in a case of megaesophagus (Giemsa—400x). (f) Myositis with granuloma in a case of megacolon (Giemsa—1000x).

Amastigotes forms of *T. cruzi* (Figure 1(a)) were identified in 4 (10%) cases of megaesophagus of a total of 39 esophagus studied. Regarding the colon, a nest of *T. cruzi* was identified only in 1 (2%) case of megacolon of 50 colon studied. *T. cruzi* nests were not detected, neither in esophagus or colon without mega. 

### 3.2. Evaluation of Inflammation in the Muscularis Propria and in the Myenteric Plexus

In cases of mega, fibrosis was observed in *muscularis propria* and myenteric plexus with a reduced number of neurons (Figure 1(b)). The inflammatory process consisted, in general, predominantly of mononuclear cells, surrounded by scattered eosinophils, few mast cells, and rare plasma cells in both muscular and plexus, either in the esophagus or in the colon, being more pronounced and frequent in megas (Figures 1(c) to 1(e)). Myositis foci with granuloma formation were identified in two cases of megaesophagus and in one case of megacolon (Figure 1(f)); in one case of megaesophagus, granulomas were found in the myenteric plexus. Generally, it was easier to find inflammatory foci in the esophagus rather than in the colon and those foci were more frequent in cases with mega compared to the ones without mega.

Table 1 shows the outcome of the myositis investigation. Myositis was observed in 27 (69%) of 39 esophagus and 23 (46%) of 50 colon, and this difference was statistically significant. Myositis was much more frequent in the organs with mega compared to those without mega, both in esophagus and colon, those differences were statistically significant. Statistical analysis also showed a higher frequency of myositis in esophagus without mega compared to colon without mega. However, although the percentage of myositis was somewhat higher in megaesophagus compared to megacolon (90% *versus* 76%), this difference was not statistically significant (Table 1).

Table 2 shows the outcome of ganglionitis investigation. Ganglionitis was very common in the esophagus, as with mega and without mega (95% *versus* 84%), having no statistically significant difference between these groups. However, ganglionitis was more frequent in chagasic patients with megacolon compared to those without megacolon (72% *versus *16%), as in the esophagus without mega compared to the colon without mega (84% *versus *16%). On the other hand, although the percentage of ganglionitis in megaesophagus has been a little higher than in megacolon (95% *versus* 72%), the difference was not statistically significant (*P* = 0.0592); however, the *P* value so close to 0.05 perhaps may suggest a trend (Table 2).

### 3.3. Characterization of the Inflammatory Infiltrate in the Muscularis Propria and Myenteric Plexus

**Figure 2:**
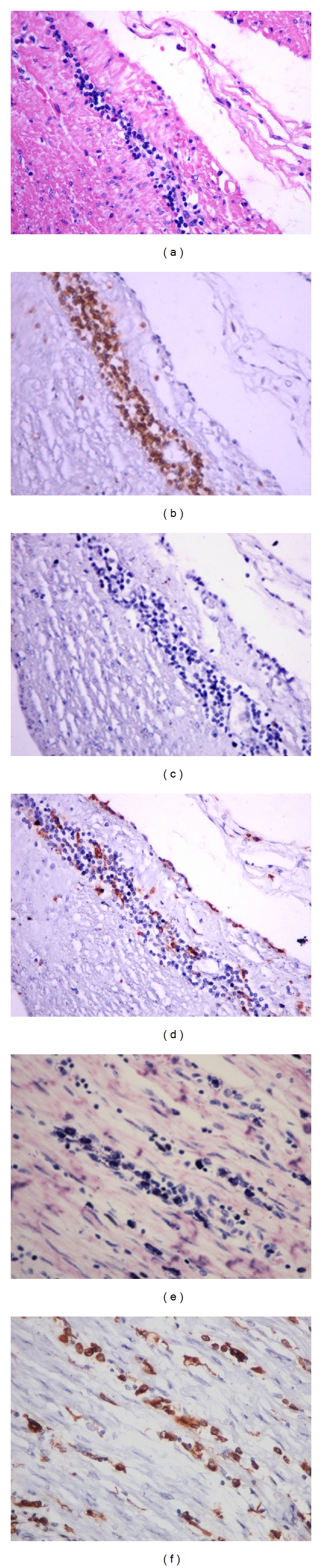
(a)–(d) Myositis in esophagus without mega. Note that the infiltrate is predominantly mononuclear ((a) HE—400x), with intense predominance of T lymphocytes ((b) immunohistochemistry for CD3—400x); extremely rare B lymphocytes ((c) immunohistochemistry for CD20—400x), and few positive CD68 cells ((d) immunohistochemistry for CD68—400x). (e) Numerous mast cells in the perimysium and in the foci of myositis in a case of megaesophagus can be seen (Giemsa—400x). (f) Compare this section stained by immunohistochemistry for CD68 with the previous figure, showing that both macrophages and mast cells are CD68 positive (400x).

After the qualitative assessment of the intensity of myositis and ganglionitis in the sections stained with HE and Giemsa, the sections stained by the immunohistochemical technique were examined in order to evaluate the predominant inflammatory cell in the foci. Among the mononuclear cells present in inflammatory foci, there was a strong predominance of CD3+ cells (T lymphocytes), in general, the CD20+ (B lymphocytes) and CD68+ (macrophages) were present in smaller numbers (Figures 2(a) to 2(d)). From the qualitative point of view, the inflammatory infiltrate was similar between the study groups; however, quantitatively, the cases with mega showed more cells in the inflammatory infiltrates. Analyzing the CD68 positive cells in the esophagus, we noticed that several of them seemed to be in the same location where we usually observe mast cells in this organ, which is known rich in these cells. Comparison between the section stained by Giemsa with that stained by immunohistochemistry for CD68 showed that several of these cells were in fact mast cells (Figures 2(e) and 2(f)). This problem was not experienced in colon, possibly because mast cells are scarce in this muscular organ.

## 4. Discussion

Concerning parasitism in the chronic phase of Chagas disease several authors emphasized its scarceness [11, 12]; but in the last decades some studies have shown that amastigotes forms of *T. cruzi* can be detected in different organs [9, 10, 13–15]. In our material we found amastigotes of *T. cruzi* only in 4 (20%) of 20 cases of megaesophagus (10% of 39 studied) and in only 1 (4%) of the 25 cases of megacolon (2% of a total of 50 chronic chagasic colon studied). Amastigotes forms of* T. cruzi *were not identified in esophagus or colon without mega. These data seem to suggest that the finding of *T. cruzi *would be less rare in organs with visceromegaly and, moreover, that in the esophagus it would be less unusual compared to the colon.

A similar result was obtained by Adad et al. [9] and Adad [10], in separate studies in esophagus and colon from chronic chagasic patients with and without mega. It is also similar to the result from Barbosa and Andrade [13], as they have detected a higher frequency of *T. cruzi* in esophagus (20%) than in colon (5%). However, it differs from these last authors, not only by the highest frequency obtained by them, but especially because we found no amastigotes in organs without mega. Maybe the difference in the frequency of cases with amastigotes compared to those identified in our study is due to the fact that those authors selected samples previously identified as positive for *T. cruzi* in routine examination of heart from chagasic cardiac patients in chronic phase or “subacute” [13], or severe inflammation in muscular tunic from esophagus [9] or colon [10]. In this study, the research of *T. cruzi* was performed in all cases, with or without inflammation. 

The frequency of positivity for *T. cruzi* in our megaesophagus cases (20%) was much lower than that obtained by Lages-Silva et al. [16], who identified “antigens deposits of *T. cruzi” *in 77% of megaesophagus, analyzing cases without previous selection and examining only small cardiomyotomy samples. This high positivity is unusual. In our study we analyzed 5 histological sections of complete rings, from each case, which represents an area of 100x greater than a specimen of cardiomyotomy. However, we highlight that in our material we consider positive only structures that had consistent morphology with amastigotes, as in immunohistochemistry are frequent artifacts and DAB precipitates that may have been interpreted as “antigenic deposits.” While knowing that the tissue parasitism is scarce in chronic phase of Chagas disease, it seems important to perpetuate the stimulus on the immune system. Studies using PCR support this hypothesis: Jones et al. [17] and Higuchi et al. [18] found, frequently, fragments of the genome of *T. cruzi* in chronic chagasic hearts; Vago et al. [19, 20] and da Silveira et al. [21] found *T. cruzi* DNA in 100% of megaesophagus cases and in 33% to 60% of esophagus without mega, as they increased the amount of tissue samples analyzed. The latter studies have used some of the cases studied in this research, indicating that although we have identified tissue forms of the parasite in a few cases using immunohistochemistry, through PCR technique it have been demonstrated persistence of the parasite in many cases. 

Regarding the inflammatory infiltrate and the presence of *T. cruzi* in histological sections of the esophagus and colon of chronic chagasic patients with and without mega, no studies were found comparing both organs simultaneously. Unfortunately, the paucity of positive cases for *T. cruzi* in our study did not enable a statistical analysis. However, myositis was more frequent in megaesophagus than in megacolon and, in the organs with mega, compared to the ones without mega, what perhaps may have some relation to parasitism.

As to the scope and frequency of inflammatory foci present in the esophagus and colon of chronic chagasic patients with and without mega, represented by myositis and ganglionitis, our results demonstrate that the inflammatory phenomena varied, being more prominent in the esophagus, especially in cases with mega. A similar comparison has not been seen in the literature. We found in the research from Barbosa and Andrade [13], who have evaluated organs without mega, mentions that the inflammatory foci were more frequent in the esophagus while in the colon they were rare or absent. In some way they could have some relationship with the higher frequency of parasitism in the esophagus to the colon. On the other hand, we might also remember that there are structural differences between the esophagus and colon: (1) there is usually greater amount of mast cells in the esophagus than in the colon, which could favor the intensification of inflammation in the esophagus [22]; (2) the presence of blood vessels in the ganglia of the myenteric plexus of the esophagus, which is not seen in the colon, could encourage primary inflammation in its ganglia [10].

Regarding the immunohistochemical characterization of the inflammatory infiltrate our results showed, in general, that the inflammatory infiltrate of the esophagus and colon from chagasic patients with and without mega are characterized by a strong predominance of T lymphocytes (CD3+), few macrophages (CD68+), and rare B lymphocytes (CD20+), regardless of the organ, the presence or absence of visceromegaly, the intensity of inflammation, and/or being ganglionitis or myositis. However, from the quantitative point of view, the organs with mega showed higher number of inflammatory cells. Similar studies were not found in the literature, which compared esophagus and colon from chagasic patients, as done in this research. Compared to isolated studies in the esophagus, our data are consistent with the literature data [23], in relation to myositis in megaesophagus; they differ, however, from these authors regarding the infiltrate in the myenteric plexus region, considering they report a predominance of macrophage-like cells CD68+ cells at this site. We believe this difference arises from the fact that we analyzed the infiltrate only within the ganglia, not considering the region around it, having that in the connective tissues around the myenteric plexus and in the perimysium there are frequent mast cells in normal esophagus and such cells are also stained by CD68 (Figures 2(e) and 2(f)).

Comparison of our results to data from other studies of colon only, it has been found in agreement with Corbett et al. [24] and da Silveira et al. [5], who also noted a predominance of T lymphocytes (CD3+) compared to B lymphocytes (CD20+) in muscular and myenteric plexus. da Silveira et al. [5] found different results in submucous plexus, where it predominated B lymphocytes (CD20+). In our study, this plexus was not analyzed for several reasons: (1) in mucosa and submucosa are very frequent changes secondary to food/stool stasis (often causing formation of lymphoid follicles), rather than being directly due to Chagas disease, as observed predominantly in muscular tunic and in myenteric plexus; (2) myenteric plexus lesions are more important for emergence of mega [25]; (3) it would not be possible to compare the changes of submucous plexus of the esophagus to the colon, considering that this plexus is almost nonexistent even in normal esophagus [9].

## 5. Conclusions

Tissue parasitism is scarce in esophagus and colon in the chronic phase of Chagas disease, being less rare in esophagus with mega. Myositis and ganglionitis are more frequent in the organs with mega when compared to esophagus without mega, and in esophagus compared to colon. While, at a qualitative point of view, the appearance of inflammatory infiltrate is similar with intense predominance of T lymphocytes (CD3+), quantitatively, the organs with mega showed higher number of inflammatory cells in both muscular and myenteric plexus.

## Figures and Tables

**Table 1:** Distribution of cases according to study groups and intensity of myositis in esophagus and colon with or without mega.

	Esophagus				Colon			
Miosytis	Without mega		With mega		Without mega		With mega	
	*n*	%	*n*	%	*n*	%	*n*	%
Present	9	47	18	90	4	16	19	76
Mild	4	21	6	30	4	16	7	28
Moderate	4	21	7	35	0	0	8	32
Severe	1	5	5	25	0	0	4	16
Absent	10	53	2	10	21	84	6	24

Total	19	100	20	100	25	100	25	100

Fisher's exact test to compare the presence or absence of myositis: esophagus × colon *P* = 0.0033, esophagus without mega × megaesophagus *P* = 0.0057; colon without mega × megacolon *P* < 0.0001; without megaesophagus × without megacolon *P* = 0.0044; megaesophagus × megacolon *P* = 0.2692.

**Table 2:** Distribution of cases according to the study groups and intensity of esophagus and colon ganglionitis.

	Esophagus				Colon			
Ganglionitis	Without mega		With mega		Without mega		With mega	
	*n*	%	*n*	%	*n*	%	*n*	%
Present	16	84	19	95	4	16	18	72
Mild	9	47	4	20	4	16	7	28
Moderate	7	37	7	35	0	0	8	32
Severe	0	0	8	40	0	0	3	12
Absent	3	16	1	5	21	84	**7**	28

Total	19	100	20	100	25	100	25	100

Fisher's exact test to compare the presence or absence of ganglionitis: esophagus × colon *P* < 0.0001; esophagus without mega × megaesophagus *P* = 0.3416; colon without mega × megacolon *P* = 0.0001; without megaesophagus × without megacolon *P* < 0.0001; megaesophagus × megacolon *P* = 0.0592.
